# A Mucosal and Cutaneous Chemokine Ligand for the Lymphocyte Chemoattractant Receptor GPR15

**DOI:** 10.3389/fimmu.2017.01111

**Published:** 2017-09-07

**Authors:** Borja Ocón, Junliang Pan, Theresa Thu Dinh, Wenjing Chen, Romain Ballet, Michael Bscheider, Aida Habtezion, Hua Tu, Brian A. Zabel, Eugene C. Butcher

**Affiliations:** ^1^The Center for Molecular Biology and Medicine, Veterans Affairs Palo Alto Health Care System and The Palo Alto Veterans Institute for Research, Palo Alto, CA, United States; ^2^Laboratory of Immunology and Vascular Biology, Department of Pathology, School of Medicine, Stanford University, Stanford, CA, United States; ^3^Lake Pharma, Inc., Belmont, CA, United States; ^4^Division of Gastroenterology and Hepatology, School of Medicine, Stanford University, Stanford, CA, United States

**Keywords:** T cells, trafficking receptor, GPR15, ligand, chemokine, colon, skin

## Abstract

Chemoattractants control lymphocyte recruitment from the blood, contributing to the systemic organization of the immune system. The G protein-linked receptor GPR15 mediates lymphocyte homing to the large intestines and skin. Here we show that the 9 kDa CC-motif containing cationic polypeptide AP57/colon-derived sushi containing domain-2 binding factor (CSBF), encoded by *C10orf99* in the human and *2610528A11Rik* in the mouse, functions as a chemokine ligand for GPR15 (GPR15L). GPR15L binds GPR15 and attracts GPR15-expressing T cells including lymphocytes in colon-draining lymph nodes and Vγ3^+^ thymic precursors of dermal epithelial T cells. Patterns of GPR15L expression by epithelial cells in adult mice and humans suggest a homeostatic role for the chemokine in lymphocyte localization to the large intestines, as well as a role in homing to the epidermis during wound healing or inflammation. GPR15L is also significantly expressed in squamous mucosa of the oral cavity and esophagus with still poorly defined regulation. Identification of the chemotactic activity of GPR15L adds to its reported antibacterial and tumor cell growth regulatory functions and suggests the potential of targeting GPR15L–GPR15 interactions for modulation of mucosal and cutaneous inflammation.

## Introduction

Chemokines are small secreted proteins that direct the systemic trafficking and microenvironmental homing of various cell types in health and disease (1). Chemokines signal through class A G protein-coupled receptors (GPCRs) and participate in homeostatic and inflammation-induced immune cell trafficking (1, 2). The orphan GPCR GPR15 directs T cell homing to the developing epidermis and to the colon and regulates colitis (3–6). GPR15 is expressed by memory B cells, plasmablasts, and memory/effector and regulatory T cell subsets (5, 7). The GPR15 polypeptidic sequence is related to that of chemokine receptors, suggesting that its physiologic chemotactic ligand might be a colon-expressed chemokine-like peptide.

Here, we describe a chemoattractant ligand (GPR15L) for GPR15, encoded by *C10orf99* in the human and *2610528A11Rik* in the mouse. The GPR15L attracts subsets of T cells in a GPR15-dependent manner. It is a 9 kDa polypeptide that has been previously described as the 57 amino acid antimicrobial peptide AP-57 (8), and as a Sushi Containing Domain-2 (SUSD2)-binding factor (CSBF), capable of inhibiting the growth of intestinal epithelial cancer cell lines (9). The GPR15L comprises a cationic disulfide-bonded domain and a conserved carboxy-terminal peptide involved in receptor activation and signaling. The chemokine is expressed by epithelial cells in gastrointestinal and genitourinary mucosae and in the skin. Expression in the colon is constitutive, developmentally determined, and only modestly affected by inflammation or the presence of microbiota. GPR15L is also expressed in squamous mucosae and is highly upregulated in skin under inflammatory settings, including psoriasis (10, 11). The GPR15L–GPR15 axis signaling may play an important role in the recruitment of specialized lymphocytes in the colon and other mucosal tissues and in sites of cutaneous inflammation. Therefore, this new ligand–receptor interaction could be of therapeutic interest for the management of autoimmune disorders of the mucosal surfaces and skin.

## Materials and Methods

### Mice and Human Subjects

GPR15^gfp/gfp^ mice from Dan Littman (4) were bred on a C57BL/6 background. Wild-type C57BL/6 mice were from JAX. Mice were maintained and bred in specific pathogen-free conditions in the animal facility at the Veterans Affairs Palo Alto Health Care Systems (VAPAHCS). Mice were used at 12–14 weeks of age, except for the neonatal thymocyte migration experiments in which day 0 pups were used. Animals were maintained in accordance to US National Institutes of Health guidelines, and experiments were approved by Stanford University Institutional Animal Care and Use Committee. Human peripheral blood mononuclear cells (PBMCs) were obtained from healthy donors. This study was carried out in accordance with the recommendations of the US National Institutes of Health guidelines, with written informed consent from all subjects. The protocol was approved by the Stanford University Institutional Review Board.

### Cell Isolation and Preparation

Thymic lymphocytes were isolated by removing the thymus and straining the whole organ first through a 100 µm mesh. Then, the first cell suspension was filtered through a 70 µm nylon mesh to obtain a single cell suspension. Caudal mesenteric lymph node (cMLN) cells were isolated from two of the colon-draining lymph nodes that are clearly separated from the chain of mesenteric lymph nodes that drain the small intestine (12). The lymph nodes were strained through a 100 µm mesh, and the cell suspension was filtered through a 70 µm nylon mesh to obtain a single cell suspension.

Human PBMCs were isolated from heparinized peripheral blood (10–40 ml) that was obtained *via* venipuncture from healthy donors. Blood samples were processed using Ficoll density gradient centrifugation (Histopaque-1077, Sigma-Aldrich). The resulting interface containing the PBMC layer was extracted, washed twice with PBS, and resuspended in the chemotaxis medium.

In all cases, after isolation cells were incubated for 2 h at 37°C/5% CO_2_ in chemotaxis medium [RPMI-1640 supplemented with heat inactivated fetal bovine serum (FBS) 10% (v/v), 100 U/ml penicillin, 0.1 mg/ml streptomycin, 2 mM glutamine, 1× MEM non-essential amino acids, and 1 mM sodium pyruvate] for receptor expression recovery, before the migration assay. Where indicated, Pertussis toxin (Tocris, Bristol, UK) treatment (200 ng/ml) was performed during these 2 h of incubation.

### Binding and Cold Competition Assay

CHO-K1 cells transfected for human GPR15 expression were from DiscoverX, Inc. (Fremont, CA, USA). We confirmed the expression and surface location of the human GPR15 in the cells by FACS using a PE-anti-human GPR15 antibody (Biolegend, San Diego, CA, USA).

In the standard-binding assays, GPR15 transfectants or normal CHO-K1 cells were incubated in binding buffer [HBSS Ca^2+^/Mg^2+^ with FBS 2% (v/v)] for 30 min with 0.1 nM of either human IgG Fc or a chimeric protein comprising the full-length human GPR15L linked to human Fc through the GPR15L N terminus (LakePharma Inc., Belmont, CA, USA).

In the “cold competition assay,” GPR15 transfectants were incubated for 30 min with several concentrations (100 pM to 10 µM) of the native full-length human GPR15L (Peprotech, Rocky Hill, NJ, USA) in binding buffer. Then the “tracer,” here human GPR15L linked to human Fc, was added to the cells at a concentration of 1 nM and incubated for 30 min.

The cells were washed in staining buffer (PBS BSA 0.5%), then stained with PE-Goat F(ab’)2 anti human IgG (Invitrogen), and acquired on a LSRII from BD, using FACS Diva Software.

### β-Arrestin Assay

CHO-K1 β-arrestin reporter cells for human GPR15 and human CMKLR1 from Discover X, Inc. (Fremont, CA, USA) were cultured normally in phenol-free RPMI-1640 supplemented with FBS 10% (v/v), 100 U/ml penicillin, 0.1 mg/ml streptomycin, 2 mM glutamine, MEM non-essential amino acids 1×, hygromycin 0.3 mg/ml, and G418 0.8 mg/ml. Briefly, cells were seeded the day before the assay in 96-well plates (Greiner bio-one, Ref:655090) at a density of 30,000 cells per well. On the day of the assay, cells were exposed for 90 min at 37°C/5% CO_2_ to different peptides in solution in the regular culture medium, then incubated for 2 h at room temperature with Tropix^®^ Gal-screen™ reagent per manufacturer’s instructions, and finally, the luminescence signal measured with an integration time of 500 ms on a SpectraMax M5 (Molecular Devices, Sunnyvale, CA, USA) plate reader. The peptides used in some of the β-arrestin assays were produced by the Stanford Protein and Nucleic Acid Facility.

### Chemotaxis Assays

*In vitro* migration assays were conducted and data presented as described (13). 1–2 × 10^6^ lymphocytes were added to the top wells of 5 µm pore, polycarbonate 24w tissue culture inserts (Costar, Cambridge, MA, USA) in 100 µl, with 600 µl of chemokine solution (or medium) in the bottom chamber. All migrations were conducted in chemotaxis medium at 37°C/5% CO_2_ for 3 h. The chemokines used were recombinant mouse and human CXCL12 (100 nM) (Gryphon Sciences, South San Francisco, CA, USA), and C10orf99 recombinant protein, Ref: NBP2-14716PEP (Novusbio, Littleton, CO, USA), here referred as human GPR15L-His. In preliminary data, we have also observed a comparable chemotactic activity for the human C10orf99 from Peprotech. The total volume of the bottom chamber of each well was harvested separately, and the migrated cells recovered were washed in staining buffer (PBS 0.5% BSA) prior to analysis by flow cytometry. Cell migration was assessed by analyzing the percentage of cells of each indicated phenotype that migrated from the top chamber to the bottom compartment for each individual insert (Figure 3). Specific migration was calculated as percentage of input cells of the indicated cell subset that migrated, minus the mean background migration of that subset in the no chemokine control group (Figures 4 and 5).

### Flow Cytometry and Antibodies

Original input and migrated cells were first blocked with staining buffer containing 10% human serum in the case of human PBMC or 1 µg/ml of anti CD16/CD32 (clone 2.4 G2, BD) for mouse cells. For intracellular Foxp3 staining, we used the transcription factor staining buffer set (00-5523-00, Thermo Scientific) following the manufacturer’s protocol. The following antibodies were used for staining of human cells: CD127-FITC (clone HIL-7R-M21, BD), Foxp3-PerCP-Cy5.5 (clone 236A/E7, BD), GPR15-PE (clone SA302A10, Biolegend), CD3-PE-Cy7 (clone SK7, BD), CLA-Biotin (559950, BD) with streptavidin-APC (BD) as secondary, CD25-APC-Cy7 (clone M-A251, BD), CD8a-Pacific Blue (clone RPA-T8, Biolegend), CD45R0-BV605 (clone UCHL1, Biolegend), and CD4-BV711 (clone SK3, BD). The following antibodies were used for staining mouse cells: CD24-PE (clone 30-F1, Biolegend), CD3e-PE-Cy7 (clone 145-2C11, BD), TCRVγ3-APC (clone 536, Biolegend), TCRγ/δ-BV421 (clone GL3, Biolegend), CD25-PerCp-Cy5.5 (clone PC61, BD), Foxp3-PE (clone MF23, BD), CD103-APC (clone M290, BD), CD44-AF700 (clone IM7, BD), CD8a-Pacific Blue (clone 56-3.7, eBioscience), CD45RB-BV650 (clone 16A, BD), CD4-BV711 (clone L3T4, Biolegend), and α4β7-APC (clone Act-1). Dead cells were excluded by DAPI, aqua dead cell staining, or fixable blue staining kit (Thermo Scientific) depending on the experiment. FACS data were acquired on a Fortessa from BD using FACS Diva software. Further analysis was performed using FlowJo from Treestar.

### RNA Isolation and Quantitative Reverse-Transcription Polymerase Chain Reaction (RT-qPCR) Analysis

Total RNA from dissected segments of the intestines was isolated using the RNeasy Mini Kit (Qiagen, Redwood City, CA, USA), according to the manufacturer’s instructions. Quantification was determined by 260/280 nm absorbance ratio; 0.5 µg RNA was subjected to reverse transcription with iScript™ Reverse Transcription kit (Biorad, Hercules, CA, USA); Power SYBR^®^ Green PCR Master Mix (Applied Biosystems, Warrington, UK) was used for amplification, and specific DNA sequences were amplified with an Applied Biosystems 7900HT sequence detection system. No PCR products were detected when the mock-transcribed mixture was used as template. Results are expressed as 2^−ddCt^ using as housekeeping genes mouse *HPRT1, RPL13a*, and *Actin*. The primers used for these housekeeping genes were commercially available primer sets from RealTimePrimers.com. The sequences of the primers for mouse *2610528A11Rik* amplification were: 5′-CAC CAC CCA TGA CTT GAC TG-3′ and 5′-CTT CTA GCC CTT TCC GGT CT-3′, purchased from Integrated DNA Technologies (Redwood City, CA, USA).

### Data and Statistical Analysis

Results are expressed as mean ± SEM. Statistics were calculated using GraphPad Prism, where differences among means were tested for statistical significance by one-way or two-way ANOVA and *a posteriori* Fisher’s least significant difference tests on preselected pairs. Differences were considered significant at *P* < 0.05.

## Results

### Chemokine-Like Features and Conservation of C-Terminal Peptide in the GPR15L

**Figure 1:**
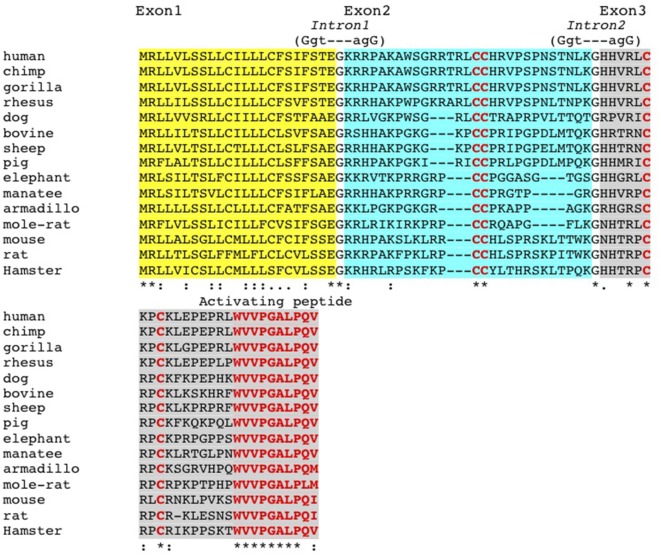
Conservation of GPR15L structure and exon/intron boundaries. GPR15L proteins from rodents to human are, respectively, encoded by three exons, and the corresponding exon/intron boundaries are illustrated in relationship to the respective protein domains. Notably, the first exon encodes the signal peptide (in italics); the second exon encodes a region enriched with positively charged residues, presumably involved in the receptor/matrix protein binding; and the third exon encodes the activating peptide (in red).

Our initial discovery efforts testing known gut- and skin-expressed chemoattractants and cytokines including CCL27, CCL28, CCL25, chemerin, and dermokine, failed to identify a binding partner for GPR15. We noted that the colon-expressed polypeptide C10orf99 was reported to trigger calcium signaling in GPR15 transfectants in a published patent application, although functional responses of lymphocytes were not evaluated (14). The *C10orf99* gene was predicted to encode a polypeptide with basic pI, low molecular weight, and structural features similar to those of known chemokines. The exon structure, position of cysteine residues (including a “CC-motif”), and the C-terminal amino acid sequence are highly conserved across vertebrate classes from human to rodents (Figure 1), consistent with a conserved function.

**Figure 2:**
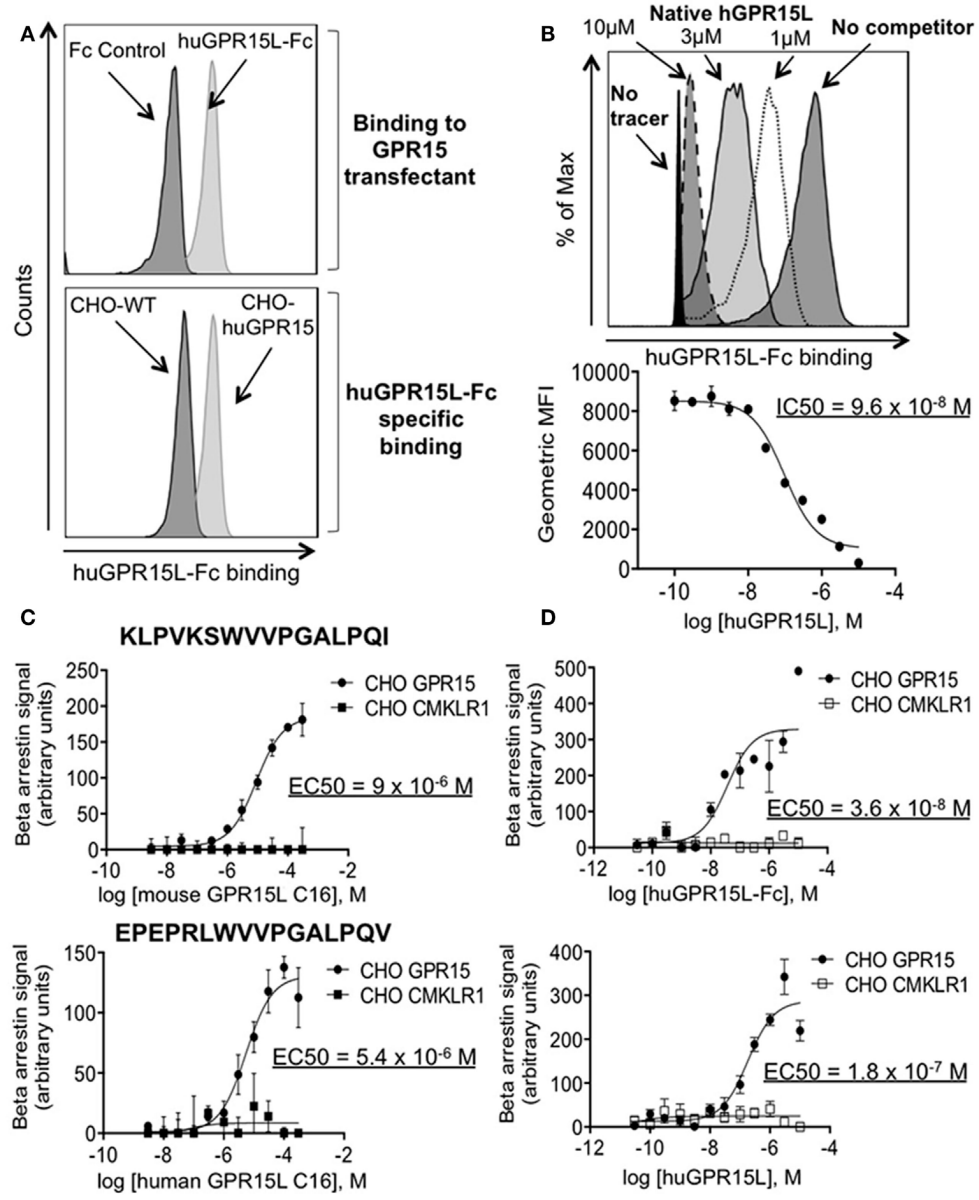
GPR15L binds specifically and is an agonist for GPR15. **(A)** Top panel; histogram showing binding of human GPR15L-Fc (0.1 nM) chimeric protein to CHO-K1 cells expressing human GPR15. Bottom panel; histogram showing specific binding of the GPR15L (0.1 nM) chimeric protein to GPR15 transfectants. **(B)** Representative histogram and graph showing the reduction in the mean fluorescence intensity (MFI) given by the human chimeric protein binding (tracer) (1 nM), due to the concentration-dependent competition of the human native GPR15L. **(C)** β-arrestin assay using CHO-huGPR15 reporter cells and CHO-huCMKLR1 as internal control, with the C-terminal peptide of the mouse GPR15L (top panel) and human GPR15L (bottom panel), containing the activating peptide. The sequences of each peptide are highlighted in bold letters on each graph. **(D)** β-arrestin assay using CHO-huGPR15 reporter cells and CHO-huCMKLR1 as internal control, with human GPR15L-Fc chimeric (top panel), and native human GPR15L (bottom panel) full-length proteins. IC50 = half maximal inhibitory concentration; EC50 = half maximal effective concentration. Data are presented as mean ± SEM, *n* = 3 for each condition and representative of three independent experiments.

We, therefore, tested the ability of the candidate GPR15L protein to bind GPR15. We linked GPR15L *via* its N-terminus to the Fc domain of human IgG. The human GPR15L-Fc chimeric protein binds huGPR15/CHO cells but not CHO controls (Figure 2A). GPR15L/GPR15 binding was inhibited by preincubation of GPR15-expressing cells with native human GPR15L, with an IC50 of 100 nM (Figure 2B). Using a β-arrestin assay to detect receptor activation, we showed that the native full length human GPR15L and the huGPR15L-Fc chimera trigger signaling with EC50’s of 20 and 40 nM, respectively (Figure 2C). Moreover, a 16 amino acid peptide corresponding to the conserved C-terminal sequence of mouse and human GPR15L was sufficient to trigger activation, albeit at higher concentrations (Figure 2D).

### GPR15L Is a Lymphocyte Chemoattractant

**Figure 3:**
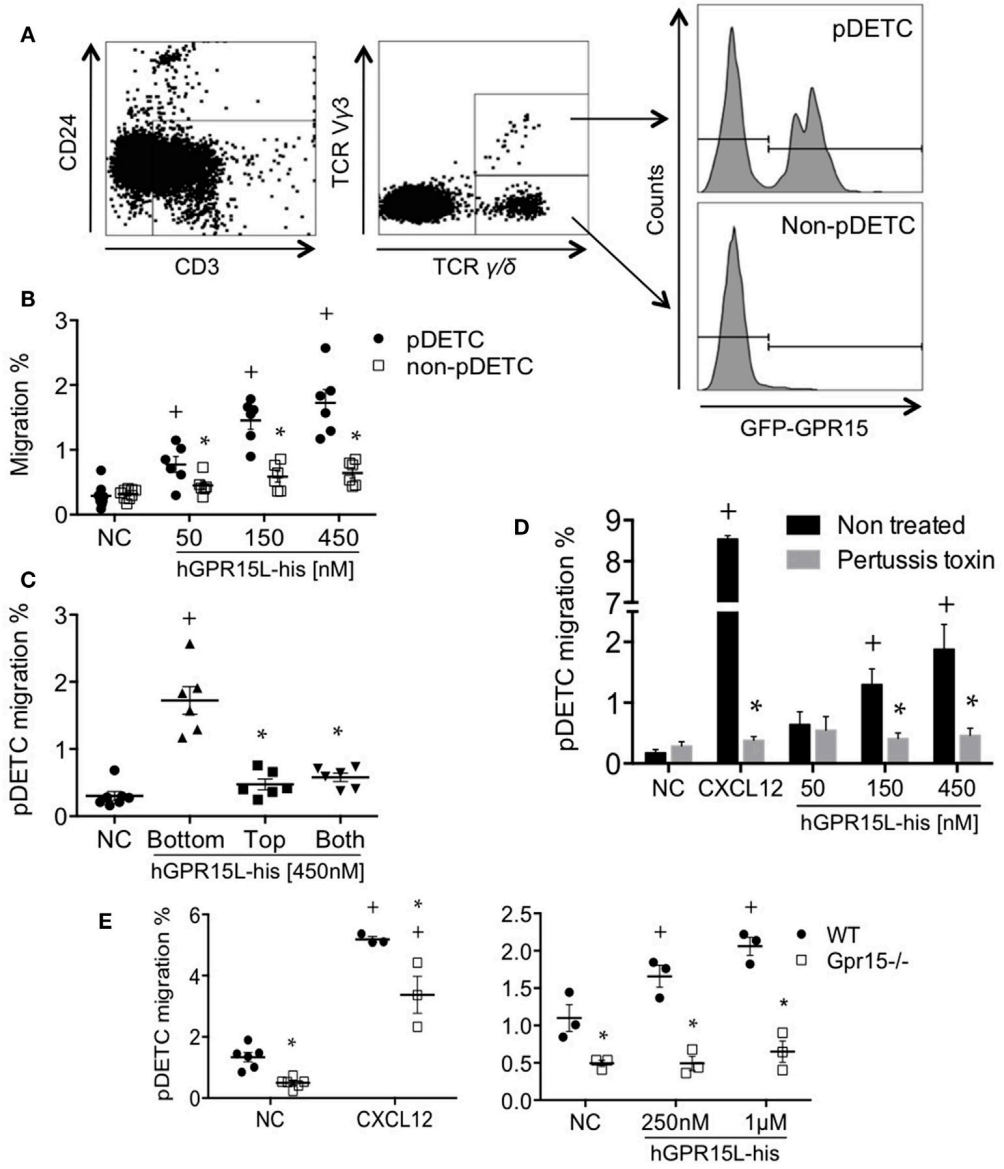
Perinatal pDETC chemotax to GPR15L in a GPR15-dependent manner. **(A)** Gating strategy and GPR15 expression of pDETC and non-pDETC in day 0 GPR15-gfp reporter mice. **(B)** Percentage of pDETC and non-pDETC cells that migrated to the bottom chamber. **(C)** Checkerboard experiment; % migration of pDETC. **(D)** Effect of pertussis toxin on the GPR15L-induced pDETC migration, expressed as % migration. **(E)** Effect of the GPR15 deficiency in the CXCL12 and GPR15L-induced migration of pDETC. Non-pDETC migrated to CXCL12 (not shown). Data pooled from two independent experiments are presented as mean ± SEM. ^+^*P* < 0.05 vs NC, *P < 0.05 vs other cell subset under the same experimental condition. DETC, dendritic epidermal T cells; NC, no chemokine; gfp, green fluorescent protein.

A subset of perinatal thymic CD3^+^ CD24^−^ TCRγ/δ^+^ TCRVγ3^+^ dendritic epidermal cell precursors (pDETC) expresses high levels of *gpr15*, and GPR15 mediates pDETC homing to the fetal epidermis (3). Using Transwell chemotaxis assays, we found that GPR15L selectively attracts pDETC, compared to their CD3^+^ CD24^−^ TCRγ/δ^+^ TCRVγ3^−^ GPR15^−^ thymic counterparts (Figures 3A,B). To determine if GPR15L increases random cell motility vs directed chemotaxis, we performed a “checkerboard” assay and found that the migration to the chemokine was predominantly chemotactic (Figure 3C). Migration was inhibited by pertussis toxin pretreatment of lymphocytes, indicating that the chemotactic effect of GPR15L is Gαi dependent (Figure 3D). Moreover, pDETC from *Gpr15*-deficient mice failed to migrate in response to GPR15L, confirming the GPR15-dependence of the chemotactic response (Figure 3E).

**Figure 4:**
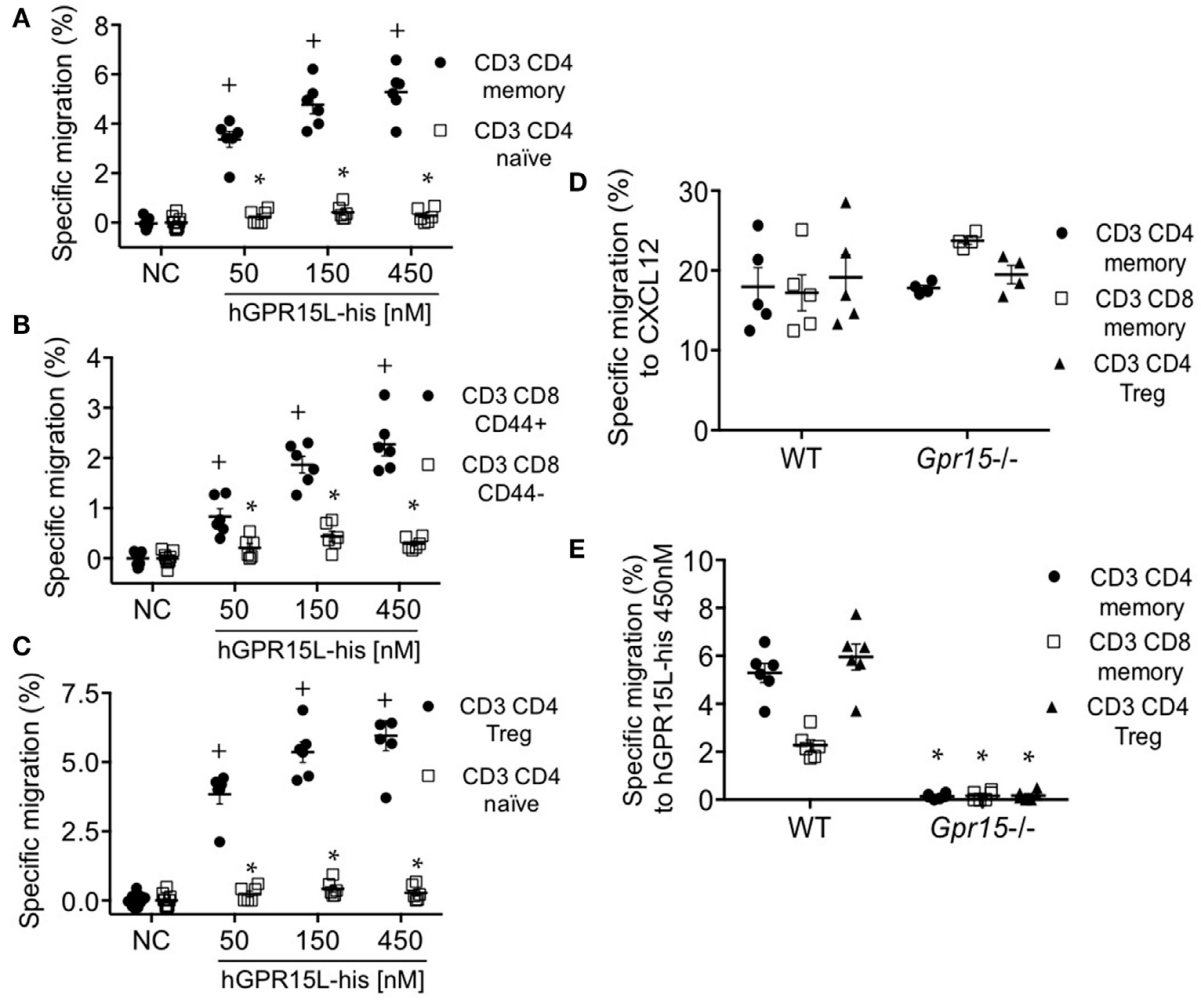
T cell subsets from colon-draining lymph nodes of dextran sodium sulfate colitic mice migrate to GPR15L in a GPR15-dependent manner. **(A)** Migration of CD4^+^ memory T cells (CD3^+^ CD4^+^ CD44^+^ CD45RB^low^ CD103^+^) and CD4^+^ naïve T cells (CD3^+^ CD4^+^ CD25^−^ CD44^−^ CD45RB^high^) to huGPR15L. **(B)** Migration of memory phenotype (CD3^+^ CD8^+^ CD44^+^) and naïve CD8 T cells (CD3^+^ CD8^+^ CD44^−^) to huGPR15L **(C)** Migration of CD4 Tregs (CD3^+^ CD4^+^ CD44^+^ CD45RB^low^ CD103^+^ CD25^+^ Foxp3^+^) compared to CD4 T naïve cells to huGPR15L. **(D,E)** Migration to mouse CXCL12 and huGPR15L of the three cell subsets that respond to the chemokine in **(A–C)**. WT and GPR15-deficient (*Gpr15*^−/−^) cells were assayed. Specific migration is given as the percent of the indicated subset that migrated minus the mean basal migration to control medium (without chemokine). Normalized data from two independent experiments with triplicates are presented, with mean ± SEM. ^+^*P* < 0.05 vs NC, *P < 0.05 vs other cell subset under the same experimental condition. NC, no chemokine.

GPR15 mediates the colon localization of CD4^+^ CD25^+^ effector and regulatory cells to the colon and has been implicated in colitis in models of infectious (4) and autoimmune (5) intestinal inflammation. Colon homing T cells are generated in the colon-draining lymph nodes, including the cMLN (12). We isolated cMLN cells from dextran sodium sulfate (DSS) colitic mice (day 7) and assessed their migration to GPR15L. CD3^+^ CD4^+^ CD44^+^ CD45RB^low^ CD25^+^ CD103^+^ memory CD4 cells and CD3^+^ CD8^+^ CD44^+^ effector memory phenotype CD8 T cells in the cMLN migrate efficiently to GPR15L, in contrast to naïve CD4 or CD8 cells (Figures 4A,B, respectively). ~80% of the migrated memory CD4^+^ T cells, and 20–30% of the migrated memory phenotype CD8^+^ T cells were α4β7^hi^ (not shown). CD3^+^ CD4^+^ Treg, which are enriched in GPR15 expression in mice (4), also migrated to GPR15L (Figure 4C). Chemotaxis of cMLN T cells to GPR15L was GPR15/*Gpr15* dependent, as T cells from *Gpr15-*deficient mice migrated normally to CXCL12, but did not respond to the new chemokine (Figures 4D,E).

**Figure 5:**
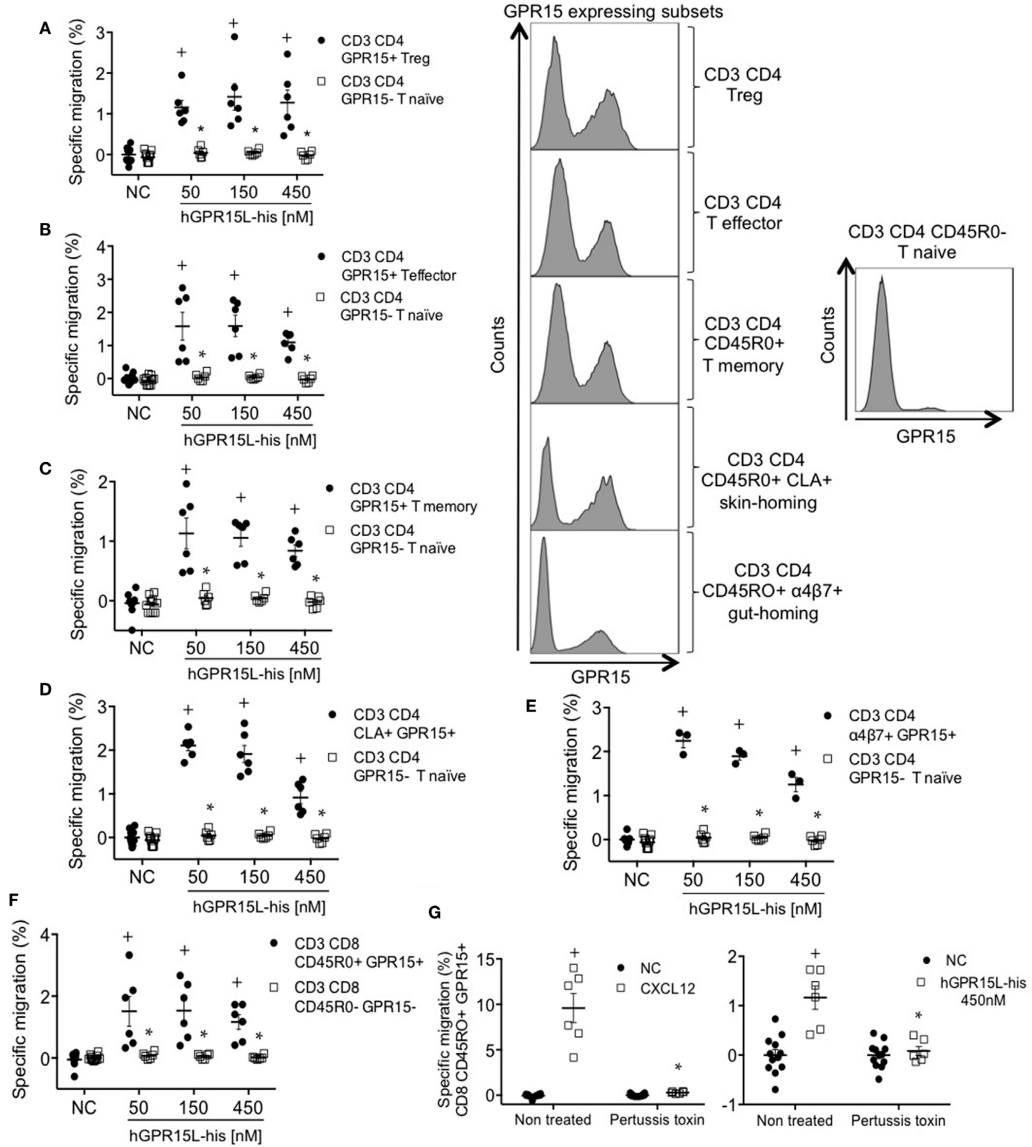
GPR15^+^ T cell subsets from human peripheral blood mononuclear cells migrate to GPR15L. **(A)** Migration of CD3^+^ CD4^+^ CD45RO^+^ CD127^−^ CD25^+^ Foxp3^+^ GPR15^+^ (CD3 CD4 GPR15^+^ Treg cells) and CD3^+^ CD4^+^ CD45RO^−^ GPR15^−^ (CD3 CD4 GPR15^−^ T naïve cells) to huGPR15L. **(B)** Migration of CD3^+^ CD4^+^ CD45RO^+^ CD127^+^ CD25^−^ Foxp3^−^ GPR15^+^ (CD3 CD4 GPR15^+^ T effector cells) and CD3 CD4 GPR15^−^ T naïve cells to huGPR15L. **(C)** Migration of CD3^+^ CD4^+^ CD45RO^+^ GPR15^+^ (CD3 CD4 GPR15^+^ T memory) and CD3 CD4 GPR15^−^ T naïve cells to huGPR15L. **(D)** Migration of CD3^+^ CD4^+^ CD45RO^+^ CLA^+^ GPR15^+^ (CD3 CD4 CLA^+^ GPR15^+^) and CD3 CD4 GPR15^−^ T naïve cells to huGPR15L. **(E)** Migration of CD3^+^ CD4^+^ CD45RO^+^ α4β7^+^ GPR15^+^ (CD3 CD4 α4β7^+^ GPR15^+^) and CD3 CD4 GPR15^−^ T naïve cells to huGPR15L. **(F)** Migration of CD3^+^ CD8^+^ CD45RO^+^ GPR15^+^ and CD3^+^ CD8^+^ CD45RO^−^ GPR15^−^ cells to huGPR15L. **(G)** Migration of CD3^+^ CD8^+^ CD45R0^+^ GPR15^+^ cells to human CXCL12 and huGPR15L. In the histograms, the GPR15 expression is shown for different GPR15-expressing CD4 T cell subsets (left) and GPR15 non-expressing CD4 T naïve cells (right). In general, data from two independent experiments were pooled, and are presented as specific migration as in Figure 4 with mean ± SEM. ^+^*P* < 0.05 vs NC, *P < 0.05 vs other cell subset under the same experimental condition. All cell subsets migrated well to human CXCL12 (data not shown). NC, no chemokine.

GPR15 is expressed by subsets of memory, regulatory, and effector lymphocytes in human blood but not on naïve T cells (7, 15). We analyzed the chemotactic effect of GPR15L on GPR15^+^ subsets of CD8 and CD4 effector/memory, Treg, CLA^+^ skin-homing, and α4β7^+^ gut-homing T cells using an anti-GPR15 antibody. GPR15^+^ subsets migrated to GPR15L, while CD4 and CD8 naïve T cells, which lack the receptor, did not respond (Figures 5A–F). Migration decreased at high concentrations, as is typical of chemotactic responses (Figures 5A–F) (16). GPR15-dependent human T cell migration to GPR15L was pertussis toxin sensitive, indicating that GPR15 couples to Gαi in human as well as mouse lymphocytes (Figure 5G).

### Tissue Expression and Regulation of the GPR15L

**Figure 6:**
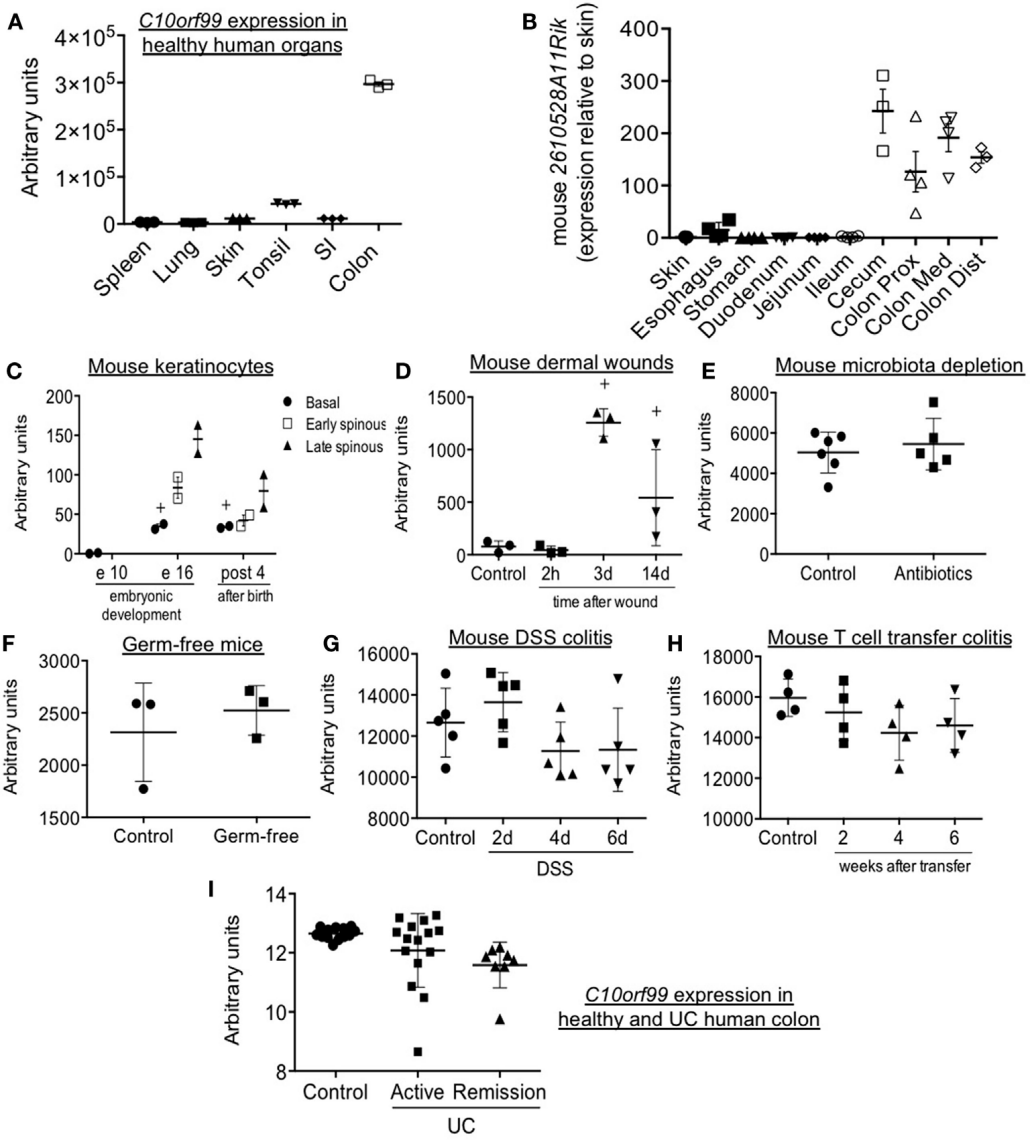
Patterns of GPR15L gene expression: mouse *2610528A11Rik* and human *C10orf99* expression in steady-state and different experimental and pathological conditions. **(A)** *C10orf99* expression in several normal human organs (18). **(B)** *2610528A11Rik* expression in skin and different segments of the gastrointestinal tract of normal C57 BL/6 mice (expressed as relative expression to that in skin). **(C)** *2610528A11Rik* expression in perinatal mouse keratinocytes (20). **(D)** *2610528A11Rik* expression in the skin of mice subjected to burn wounds. **(E)** *2610528A11Rik* expression in the colon of mice with acquired microbiota depletion by treatment with antibiotics in drinking water (21). **(F)** *2610528A11Rik* expression in the colon of germ-free mice (22). **(G)** *2610528A11Rik* expression in the colon of DSS colitic mice at different time points (23). **(H)** *2610528A11Rik* expression in the colon of T cell transfer colitic mice at different time points (24). **(I)** *C10orf99* expression in the colon of healthy subjects and patients of ulcerative colitis both in active and remission condition (25). Except in panel B (RT-qPCR data), the RNA-seq gene expression data are from the NCBI gene expression repository.

Previous studies have reported tissue-selective expression of *C10orf99* and *2610528A11Rik*, with highest gene and protein expression in the colon and stomach mucosa, and substantial expression in the oral cavity and genitourinary tract as well (17). We confirmed selective expression in human large intestine by analysis of independent data sets in the NCBI gene expression repository (17, 18) (Figure 6A). In addition, we have confirmed a comparable pattern of expression in the mouse, with selective and high expression of *2610528A11Rik*/*GPR15L* in the distal segments of the gastrointestinal tract (cecum and colon) (Figure 6B). In these sites, GPR15L appears restricted to epithelial cells (Human Protein Atlas available from www.proteinatlas.org). The colonic expression of GPR15L is developmentally programmed, since the gene is already expressed in the caudal endoderm by day 9.5–13 of embryonic development (19).

As mentioned above, pDETC migrate from thymus to epidermis in a GPR15-dependent manner between day 16 and 18 of embryonic development (3). Consistent with this, by analyzing public data sets of the NCBI gene expression repository, we found that the *2610528A11Rik* is highly expressed in keratinocytes of embryonic (day 16) and neonatal skin. Expression is highest in the spinous layer, which harbors recruited DETC (20) (Figure 6C). In contrast, GPR15L message is low or absent in adult skin in both mouse and human (Figures 6A,C); but it is significantly increased during wound healing, peaking 3 days after a burn injury (Figure 6D). Moreover, in humans, *C10orf99* is highly upregulated in psoriasis (10, 11).

Analyzing public data sets of the NCBI gene expression repository, we have observed that colonic expression of *2610528A11Rik*/*GPR15L* is not altered by intestinal microbiota (Figures 6E,F) (21, 22). It is only minimally affected by intestinal inflammation, since it is not significantly modulated in two classic experimental models of colitis like the DSS and the T naïve cell transfer model (Figures 6G,H) (23, 24). In line with this, in humans, *C10orf99*/*GPR15L* colonic expression is not significantly altered by inflammation in the context of ulcerative colitis (UC) (Figure 6I) (25).

## Discussion

Our results demonstrate that GPR15L binds to GPR15 and functions as a potent chemoattractant for GPR15-expressing lymphocytes in mouse and human. We and others, have previously shown that GPR15 mediates lymphocyte homing to the colon, and consistent with this we show that effector/memory and regulatory lymphocytes in colon-draining lymph nodes, a site where cells are educated and imprinted for trafficking to the large intestine (12), migrate well to GPR15L. We also show that Foxp3^+^ (Treg), CLA^+^ (skin-homing), and α4β7^+^ (gut-homing) GPR15^+^ CD4, as well as CD8 effector T cell subsets in human PBMC migrate specifically to GPR15L. Gene and protein expression studies indicate that GPR15L is constitutively expressed in the colon, likely from early development; and its expression in the colon is only minimally influenced by inflammation. These patterns are consistent with a prominent role of GPR15L in the GPR15-dependent lymphocyte homing to the colon, both in steady-state and in disease. As GPR15 mediates colitis in T cell-dependent mouse models (5) and is highly expressed on presumptive pathogenic Th2 cells in the *lamina propria* of patients with UC (5), our results suggest the GPR15L–GPR15 interaction may prove useful as a therapeutic target for inflammatory diseases of the large intestines.

The findings also support an important role for GPR15L–GPR15 interaction in the skin. pDETC, a thymic cell subset that express GPR15 and migrate to the epidermis in a receptor-dependent manner, migrate specifically to GPR15L. DETC play a prominent role in establishing and maintaining the barrier function of cutaneous epithelium in the developing mouse (26), and they promote keratinocyte proliferation during wound healing in adult mice as well (27). In this context, it is interesting that GPR15L, while highly expressed in fetal and neonatal epidermis at the stage of DETC recruitment in the mouse, is nearly absent in the uninflamed adult epidermis; but is highly upregulated in settings of wound healing in the mouse, and in psoriasis in the human. Indeed, GPR15L/*C10orf99* expression is a useful feature for classification of the disease (10). Taken together, these considerations suggest that in the skin, GPR15L–GPR15 interaction may recruit effector lymphocytes in settings requiring epithelial repair when a protective immune and wound healing response is needed, and potentially in infection where the antimicrobial activity of the ligand may also play a role.

In this context, it is interesting that local sustained delivery of GPR15L promotes granulation tissue formation and wound healing in a full-thickness dermal defect rat model (28). Similarly, transgenic overexpression of GPR15L seems to confer significant protection in the imiquimod-induced model of psoriasis (14). Interestingly, as in skin wound healing (Figure 6D), the GPR15L is markedly upregulated in that experimental mouse model of psoriasis (our data, not shown). Whether GPR15-dependent recruitment of immune cells contributes to these “healing” activities of GPR15L remains to be determined.

The GPR15L gene (*C10orf99* or *2610528A11Rik*) and/or protein (immunohistology) are also expressed in the esophagus, stomach (fundic gland in the mouse), bladder, cervix, cornea of the eye, and testis (8). Although the ligand is easily detectable by immunohistochemistry (especially in testis), in general, it is as yet unclear whether that GPR15L expression is constitutive or regulated in these sites. Interestingly, while GPR15L expression in testis is very low or absent at birth and during development, it becomes quite significant when mice mature (10 weeks) (https://gtexportal.org and http://biogps.org).

GPR15L lacks significant homology to classic CC and CXC chemokines, yet shares many features with them, including a cationic domain predicted to comprise two disulfide bonds and a highly conserved GPCR-activating terminal peptide (Figure 1). The activating peptides of CC chemokines are typically located at the N-terminus, while that of GPR15L is C-terminal, a feature that it shares with some other GPCR ligands like chemerin and apela. Interestingly, GPR15 shares homology with the chemerin receptor CMKLR1, and is more similar in putative ligand-binding sequences to CMKLR1 and the apelin receptor than to receptors for the classical chemokines.

The structure of GPR15L, as in other chemokines, may allow two-step, dual-site binding as originally described for C5a (29). In this model, charge-dependent interactions between the negatively charged N terminus of GPR15 and the cationic N-terminal domain of GPR15L would initiate the ligand–receptor binding. Tyrosines in the GPR15 N-terminus may be sulfated (30), potentially enhancing the charge-based recruitment of the cationic domain of GPR15L to the receptor. Interaction would be followed by insertion of the free hydrophobic GPR15L C-terminal amino acids into the signaling pocket of the receptor.

The new chemokine and its receptor may also have other functions. Future studies will show whether GPR15–GPR15L interaction triggers rapid integrin activation and/or regulates cell proliferation like many chemokines. The receptor may have other ligands as well (31). The ligand may also have GPR15-independent receptors and functions. Indeed, similar to the mucosal chemokine CCL28 and the skin inflammation-associated cathelicidin peptide LL37 (ligand of the GPCR formyl peptide receptor 2 and others) (32), at micromolar concentrations, GPR15L has broad antimicrobial activity against different organisms, including Gram-positive *Staphylococcus aureus, Actinomyce*, and fungi *Aspergillys niger* as well as *Mycoplasma* and lentivirus (8). GPR15L is also a high affinity ligand for SUSD2, and co-expression of SUSD2 and GPR15L inhibits growth of some cancer cell lines by inducing G1 arrest (9). Mice deficient in *2610528A11Rik* encoding GPR15L, generated as part of a large scale study of mutants of secreted proteins, showed a slight growth defect as well as a significant increase in blood CD4/CD8 T cell ratio and decrease in serum IgM (33). Whether the chemotactic activity of GPR15L described here contributes to its reported effects on skin wound healing and systemic immunologic parameters remains to be determined.

## Ethics Statement

Animals were maintained in accordance to US National Institutes of Health guidelines, and experiments were approved by Stanford University Institutional Animal Care and Use Committee. Human peripheral blood mononuclear cells were obtained from healthy donors. This study was carried out in accordance with the recommendations of the US National Institutes of Health guidelines, with written informed consent from all subjects. The protocol was approved by the Stanford University Institutional Review Board.

## Author Contributions

BO performed experiments and wrote the manuscript. WC and HT produced full-length peptides. BZ, WC, and HT designed and produced the human GPR15L-Ig Fc. TTD, RB, JP, and BZ screened chemokines. MB provided technical expertise and methodological support. JP analyzed the evolution and predicted properties of the chemokine. BZ supervised work and BZ and JP edited the manuscript. AH provided tissues and support. EB initiated and supervised the research and wrote the manuscript.

## Conflict of Interest Statement

The authors declare that the research was conducted in the absence of any commercial or financial relationships that could be construed as a potential conflict of interest.
